# Stability Studies of the Vaccine Adjuvant U-Omp19

**DOI:** 10.1016/j.xphs.2020.10.011

**Published:** 2021-02

**Authors:** M. Laura Darriba, María L. Cerutti, Laura Bruno, Juliana Cassataro, Karina A. Pasquevich

**Affiliations:** a Instituto de Investigaciones Biotecnológicas (UNSAM-CONICET), Universidad Nacional de San Martín, Buenos Aires, Argentina; b Fundación Instituto Leloir, IIBBA-CONICET, Buenos Aires, Argentina

**Keywords:** Vaccine adjuvants, Stability, Analytical biochemistry, Protein formulation, Protein aggregation, Preformulation, Circular dichroism, Light scattering (dynamic), Lyophilization, Mucosal immunization, Oral drug delivery, Mucosal vaccination, Protease, Physicochemical properties

## Abstract

Unlipidated outer membrane protein 19 (U-Omp19) is a novel mucosal adjuvant in preclinical development to be used in vaccine formulations. U-Omp19 holds two main properties, it is capable of inhibiting gastrointestinal and lysosomal peptidases, increasing the amount of co-administered antigen that reaches the immune inductive sites and its half-life inside cells, and it is able to stimulate antigen presenting cells *in vivo*. These activities enable U-Omp19 to enhance the adaptive immune response to co-administrated antigens.

To characterize the stability of U-Omp19 we have performed an extensive analysis of its physicochemical and biological properties in a 3-year long-term stability study, and under potentially damaging freeze-thawing and lyophilization stress processes. Results revealed that U-Omp19 retains its full protease inhibitor activity, its monomeric state and its secondary structure even when stored in solution for 36 months or after multiple freeze-thawing cycles. Non-enzymatic hydrolysis resulted the major degradation pathway for storage in solution at 4 °C or room temperature which can be abrogated by lyophilization yet increasing protein tendency to form aggregates.

This information will play a key role in the development of a stable formulation of U-Omp19, allowing an extended shelf-life during manufacturing, storage, and shipping of a future vaccine containing this pioneering adjuvant.

## Introduction

Oral immunization offers several advantages over other routes of administration.^1^, ^2^, ^3^ Mucosal vaccines are highly effective providing both mucosal as well as systemic immunity.^1^, ^2^, ^3^, ^4^, ^5^ This route of administration presents logistical and regulatory advantages over parenterally given vaccines and may be the preferred choice for emerging pathogens or the need of mass vaccination in case of a pandemic or epidemic. However, safety and regulatory issues remain to be attended; since the few current licensed oral vaccines are live attenuated or non-living whole-cell vaccines.^1^^,^^2^^,^^4^ In this light, subunit vaccines lacking entire pathogens are considered to be safer.^2^ Notwithstanding, some fundamental considerations must be upheld when developing subunit mucosal vaccines, including poor immunogenicity, degradation of its constituents in the harsh mucosal environment, delivery of its materials to mucosal immune inductive tissue, and modulation of the mucosal immune environment such that oral tolerance does not develop.^1^, ^2^, ^3^, ^4^ These obstacles will be overcome by developing effective mucosal adjuvants.^1^^,^^3^^,^^5^

Adjuvants are molecules or compounds that enhance the immune response to co-administered antigens (Ags) after immunization. The major limiting factor for oral vaccine development is the restricted availability of mucosal adjuvants, therefore there is a clinical need for new adjuvants to enhance and broaden immune responses of various vaccine candidates.^1^, ^2^, ^3^, ^4^, ^5^, ^6^ The best-studied mucosal adjuvants are the bacterially derived ADP-ribosylating enterotoxins. This family, that includes heat-labile enterotoxin of *Escherichia coli* (LT) and cholera toxin (CT), promotes a multifaceted antigen-specific response. However, toxin-based adjuvants present highly toxic or reactogenic effects in humans, even when administered in minimal doses. Nevertheless, protein engineering strategies reduced their toxicity through the development of mutants or subunits. The most promising enterotoxin-based adjuvant to date, LT(R192G/L211A) known as double mutant LT (dmLT) maintains the immunostimulatory properties of the parental LT without the associated epithelial cell toxicity or intestinal fluid secretion.^7^^,^^8^ Until the present, there are no marketed oral vaccines containing any of these adjuvants.

Nowadays, there is an urgent need to develop new oral adjuvants, especially those able to induce T helper 1 (Th1) and CD8^+^ T cell responses and prevent infectious diseases related to intracellular pathogens.^5^ In this regard, our laboratory has been extensively working on the use of the *Brucella* spp. unlipidated outer membrane protein 19 (U-Omp19) as an adjuvant. U-Omp19 is a novel protein adjuvant that is in preclinical development with various vaccine candidates to be used mainly in oral delivered-, but also in parenteral delivered formulations. We have demonstrated its ability to strengthen the immune response against different co-delivered antigens of diverse origins (bacterial, parasitic, viral, tumoral) by enhancing Th1, Th17, cytotoxic T lymphocytes (CTLs), and antibody responses.^9^, ^10^, ^11^ U-Omp19 is able to stimulate cells of the immune system and also, it is capable of partially inhibiting host gastrointestinal and endosomal proteases. These two main properties may explain its adjuvant activity by protecting co-delivered antigens from degradation, increasing their half-life, promoting their arrival to induction sites and enhancing the elicited antigen-specific immune response.^9^, ^10^, ^11^, ^12^, ^13^

To be used as a drug, proteins must be stable during development, production and storage, preserving both activity and structure. Since vaccines are prone to physical and chemical degradation during manufacturing, storage, and/or delivery, a careful analysis of the stability-influencing factors may help to avoid or mitigate these problems.^14^, ^15^, ^16^ Unfolding, aggregation and chemical modifications are the most common protein degradation pathways that can lead to a loss of the native conformation. Thus, identifying any factor affecting the quality and stability of vaccine components is of utmost importance to prevent undesired degradation reactions over time, which can ultimately lead to a drop of its biological potency.^17^

In the last years, we have gained much knowledge about U-Omp19's adjuvant qualities, however, less is known about its physicochemical properties as well as its stability (both, structural and functional). Structural characterization, stability studies and formulation development of U-Omp19 will play a key role in its successful process development, scale up, commercial manufacturing and storage. Thus, the aim of the present work was to investigate the stability of this new mucosal adjuvant when stored under a broad range of conditions as well as to establish a battery of analytical assays to detect and identify any early change in the protein. Coming across with methods that fit for this purpose will be crucial not only during the development phase of U-Omp19 containing products, but also for in process quality control tests during production, and to conduct comparability and batch stability studies.

As biophysical methods are the principle techniques used to determine if protein products have the appropriate higher order structure and can maintain the active conformation, we have used Far – UV circular dichroism (Far – UV CD), dynamic light scattering (DLS), size-exclusion chromatography (SEC) and sodium dodecyl sulfate-polyacrylamide gel electrophoresis (SDS-PAGE) to evaluate U-Omp19 stability under a variety of storage conditions. In addition, the protease inhibitor activity of this adjuvant vaccine candidate has been followed during the entire study as an indicator of its biological function.

## Materials and Methods

### Protein Purification

Recombinant U-Omp19 was expressed in *E. coli* BL21(DE3) and purified by immobilized metal ion affinity chromatography (IMAC) as previously described.^18^ An additional size-exclusion chromatography coupled to fast protein liquid chromatography purification (SEC-FPLC) step was included for samples used for long-term storage studies and for samples subjected to freeze–thaw cycles and freeze-drying. Briefly, the IMAC eluted protein was concentrated using Amicon Ultra centrifugal (3000 Da MW cut-off) filter (Amicon, Millipore) and further purified on a Superdex-75 column (GE Healthcare) with isocratic elution in gel filtration buffer (50 mM NaH_2_PO_4_, 300 mM NaCl, pH 7.4). Purified protein samples were filtered through a 0.22 μm Millex-GP filter membrane and protein concentration was determined (see below). Samples quality was checked by 15%-SDS–PAGE. Final protein stocks ranged from 1.5 to 2.5 mg/mL.

### Analytical Methods

#### Protein Quantification

##### UV Absorbance

Protein concentration was determined spectrophotometrically in a UV–VIS spectrophotometer (Beckman Coulter DU 530, Beckman Instruments Inc., California, USA). Five hundred microliters of an approximately 0.5 mg/mL U-Omp19 solution were prepared. The mean of three absorbance spectra was recorded in the region between 220 and 320 nm (1.0 nm steps) and the sample protein content was determined based on the absorbance at λ = 280 nm and the dilution factor applied. The theoretical molar extinction coefficient of the purified protein (*ε*_280_ = 16,960 M^−1^ cm ^−1^) was estimated from its sequence using the ProtParam tool from the ExPASy server.^19^

##### Bicinchoninic Acid Assay (BCA)

Protein content analysis by BCA was performed using the commercial Pierce™ Micro BCA Protein Assay Kit (Thermo Fisher, USA) following the supplier's instructions. Absorbance was measured at 562 nm using a 96-well microplate spectrophotometer (FilterMax F5, Molecular Devices, USA) with BSA as standard.

#### α-Chymotrypsin Inhibitor Activity

U-Omp19 protease inhibitor activity was measured as the reduction in the rates of α-Chymotrypsin mediated cleavage of specific fluorogenic substrate, Suc-Ala-Ala-Pro-Phe-AMC, (Calbiochem, California, USA) as previously described.^10^ Briefly, α-Chymotrypsin (1.5 nM, Sigma-Aldrich) was incubated in the presence or absence of U-Omp19 (50 μM) for 1 h at RT in black, half-area, flat-bottom, 96-well polystyrene plates (Corning Inc., New York, NY, USA). Subsequently, 60 μM substrate solution was added to each well and afterwards substrate fluorescence emission was measured kinetically with a fluorometer (FilterMax F5, Molecular Devices, USA) using the 360 nm and 465 nm excitation and emission filters, respectively.

#### Size Exclusion Chromatography (SEC)

Samples were analyzed by isocratic size exclusion FPLC with UV detection using a Superdex75 10/300 GL column (GE Healthcare, USA). The mobile phase contained 300 mM NaCl in 50 mM Tris-HCl at pH 8.0. The flow rate was 0.5 mL/min and detection was set at 280 nm. Protein mass loaded was 200 μg with an injection volume of 500 μL.

#### Dynamic Light Scattering (DLS)

Size and aggregation state were monitored by DLS using a Zetasizer Nano-S (Malvern Instruments, UK). After being spectrophotometrically quantified, samples were diluted to a final concentration of 1.0 mg/mL in 50 mM Tris-HCl, 300 mM NaCl, pH 8.0 buffer. Samples were analyzed at 25 °C. Scattered light was detected at an angle of 173° (backscatter) and three measurements consisting of 10 runs each (10 s per run) were conducted per individual sample. Protein parameters were analyzed with the DTS v.7.11 software provided by the supplier. Polydispersity index (PdI) for the individual peaks in the Intensity distribution were calculated as $PdI={\left(\frac{\sigma }{d}\right)}^{2}$, where σ represents the standard deviation and *d* is the mean value of the hydrodynamic diameter of an individual peak.^20^

#### Far- UV Circular Dichroism (Far- UV CD)

A J-815 spectropolarimeter (JASCO, Tokyo, Japan) was used to analyze the secondary structure content. After being spectrophotometrically quantified, samples were diluted to 10 μM in 10 mM NaH_2_PO_4_ pH 7.0 buffer. Spectra were recorded in the far UV region from 260 to 197 nm at 25 °C in a quartz cell of 0.1 cm pathlength, with a scan speed of 100 nm/min and a bandwidth of 4 nm. Each spectrum was the mean of 3 accumulated scans.

#### SDS-PAGE and Densitometric Analysis

U-Omp19 samples were run under both non-reducing and reducing conditions by SDS-PAGE. Samples were mixed with 5 × sample buffer (60 mM Tris pH 6.8, 70 mM SDS, 0.1 mM tetrabromophenol blue and 35% glycerol diluted in water) to a final concentration of 1 ×. For reduced samples, 50 mM β-Mercaptoethanol was added. The samples were incubated at 95 °C for 5 min. Twenty μg of U-Omp19 protein were analyzed by SDS-PAGE on a 15% gel using Tris-Glycine running buffer (25 mM Tris, 192 mM glycine, 0.1% sodium dodecyl sulfate, pH 8.3). Protein bands were visualized by staining with Coomassie blue R250 and destained with 40% methanol and 10% acetic acid solution. High resolution images of scanned Coomasie-blue stained gels were imported into ImageJ software (NIH, Bethesda, Maryland, USA). The individual lanes were selected and the profiles of the plot were obtained, mean pixel density and total area under the curve (AUC) were measured and values were used for further analysis. Percentage of intact protein was calculated as the ratio of the band intensity of U-Omp19 versus the total AUC.

### Stability Studies

#### Long-Term Storage Stability Study

Two mL cryotubes (Corning Inc., USA) were aseptically filled with 0.3 mL of a 2.3 mg/mL U-Omp19 solution and tightly sealed. Aliquots were stored in opaque boxes at 20 °C, 4 °C, −20 °C or −80 °C and pulled out for analysis at initial time, 24 h, and at 1, 2, 3, 6, 9, 12, 18, 24 and 36 months respectively. At each time point samples were centrifuged at 13,000×*g*, 4 °C for 10 min and the supernatant was analyzed. Before and following centrifugation, samples were inspected visually by naked eye to look for any visible particles or pellet in the tube. After centrifugation supernatants were placed into new tubes. The analytical assays included determination of total protein content, size and aggregation state by SEC-FPLC, DLS and SDS-PAGE, secondary structure content by Far-UV CD and U-Omp19 protease inhibitor activity (see below).

#### Freeze–Thawing Stability Analysis

Aliquots (0.5 mL) of U-Omp19 (1.5 mg/mL) solution were pipetted into polypropylene tubes which were placed at −80 °C for 24 h. Samples were then thawed at room temperature for 2 h and frozen again at −80 °C once per day up to 6 times. After being subjected to cycles of freeze and thaw, each sample was centrifuged at 13,000×*g* at 4 °C for 10 min, and the supernatants were assessed for protein content, protease inhibitor activity, non-reducing SDS-PAGE and DLS. Before and following centrifugation, samples were inspected visually by naked eye to look for any visible particles or pellet in the tube. After centrifugation supernatants were placed into new tubes.

#### Freeze-Dry Experiments

Polypropylene tubes were filled with 500 μL of the U-Omp19 stock solution (2.5 mg/mL). After being frozen for 24 h, samples were lyophilized for 24 h on a freeze dryer (Labconco Freeze Dry System, Kansas, MO, USA). Dried samples were stored at RT, 4 °C, and −80 °C for 1 week, or 1, 2, 3, 6 and 18 months and rehydrated with high purity water. Samples were centrifuged at 13,000×*g*, 4 °C for 10 min and the supernatant was analyzed. Before and following centrifugation, samples were inspected visually by naked eye to look for any visible particles or pellet in the tube. After centrifugation supernatants were placed into new tubes. Protein concentration, protease inhibitor activity, protein integrity and aggregates formation were analyzed after reconstitution at the indicated time points.

### Statistical Analysis

GraphPad Prism 7 software (GraphPad, San Diego, CA) was used for Statistical analysis and plotting. One-way ANOVA test followed by the Bonferroni multiple-comparison post-test was used to analyze differences among storage conditions in results of residual protease activity assays and percentage of undigested protein at each evaluated time point. Linear regression analysis of percentages of residual activity versus storage time, Rh versus storage time or PdI of main peak versus storage time at each storage condition were performed and then slopes were tested for significant differences to zero (P < 0.01) using a F test. Data of percentage of undigested protein versus time were fitted to one phase decay equation model to compare the behavior of samples. Best fit values were used to estimate the initial rates of degradation and the half-life for each condition. The goodness of fit is depicted as R square. An extra sum-of-squares F test was applied to compare if fitted parameters are statistically different for each condition. To achieve reproducibility, all experiments were performed two or three times.

## Results

### U-Omp 19 Stability in Long Term Storage Conditions

Storage conditions can affect the inherent structural complexity and marginal stability of protein-based antigens or adjuvants or pharmaceutical products. Major structural changes generally occurring under non-optimized storage conditions are aggregation or non-enzymatic proteolysis, which may affect the efficacy and/or safety of the product. Thus, to determine the stability of protease inhibitor activity, tendency to form aggregates, loss of folding or structural integrity of U-Omp19 during long-term storage, an extensive analytical characterization of its biochemical and physicochemical properties under different storage conditions was carried out. Protein samples in phosphate buffer were stored frozen at −20 °C or −80 °C or as a liquid formulation at 4 °C or 20 °C -room temperature (RT)-. At different time points samples were analyzed for protein concentration, protease inhibitor activity, presence of aggregates, secondary structure content and integrity. Neither protein precipitation nor changes in visual appearance (color/opacity) were observed in any sample along the 36-month study (data not shown). Protein concentration of frozen stored samples remained stable along the entire experiment. However, samples stored as liquid formulations at RT and 4 °C showed an increment in protein concentration after 1.5–2 years of storage, a phenomenon probably linked to solvent evaporation (Supplementary Fig. 1).

### U-Omp19 Retains its Full Protease Inhibitor Activity When Stored in Solution at Different Temperatures up to 36 Months

**Fig. 1:**
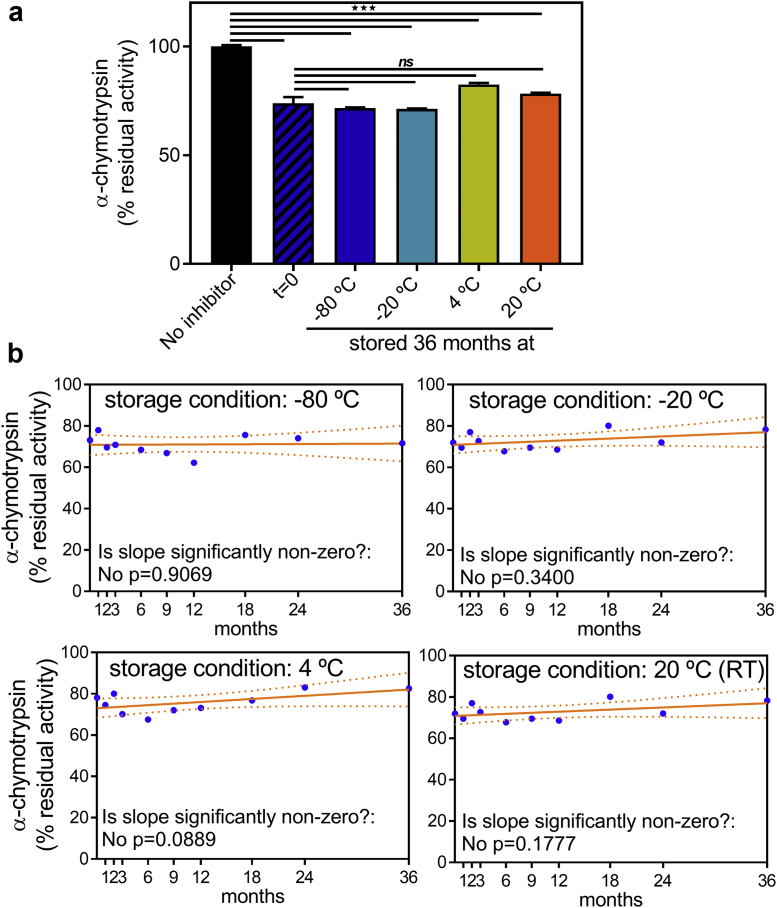
Impact of long-term storage on U-Omp19 protease inhibitor activity. Protease inhibitor activity was determined by an α-Chymotrypsin activity assay using a specific fluorogenic substrate in which the increment in fluorescence is proportional to proteolytic activity. α-Chymotrypsin was pre-incubated for 1 h with buffer (No inhibitor) or 50 μM of each U-Omp19 sample: fresh prepared (t = 0), or stored for different time periods (1 day or 1, 2, 3, 6, 9, 12, 18, 24 and 36 months) at the indicated temperatures (−80 °C, −20 °C, 4 °C, and 20 °C). Inhibitor activity is expressed as percentage of protease activity remaining when compared to “No inhibitor” condition (100% of activity). (a) Representative bar graph for the protease inhibitor activity analysis at time point = 36 months. Each bar represents the mean residual proteolytic activity of α-chymotrypsin and error bars represents SEM. ★★★: P < 0.001 vs No inhibitor condition and ns: P > 0.05 vs t = 0 condition. (b) Percentages of α-Chymotrypsin residual activity (mean ± SEM) were plotted versus time of storage at each temperature: −80 °C, −20 °C, 4 °C and 20 °C. Regression lines and 95% confidence intervals are shown for each condition. Fitted slopes were compared to 0 (No changes in time) using an F test and P values are depicted on each graph.

To evaluate the stability of U-Omp19's protease inhibitor activity, its ability to inhibit α-chymotrypsin was determined monthly during the first year, every 6 months during the second year and yearly until the third year of storage in samples kept in solution at different temperatures. Remarkably, the ability of U-Omp19 to inhibit α-chymotrypsin remained very stable along the entire study in all storage conditions tested (Fig. 1a). The regression lines of protease inhibitor activity versus time were found to be parallel and the common slope was not significantly different from zero (p > 0.05), indicating that no significant loss of inhibitor activity occurred in any of the tested conditions (Fig. 1b). Together these results indicate that the protease inhibitor activity of U-Omp19 is stable for up to 3 years, remaining unaffected when stored in solution at −80 °C, −20 °C, 4 °C or RT.

### U-Omp19 Does Not Aggregate Nor Lose its Secondary Structure When Stored in Solution at Different Temperatures up to 36 Months

**Fig. 2:**
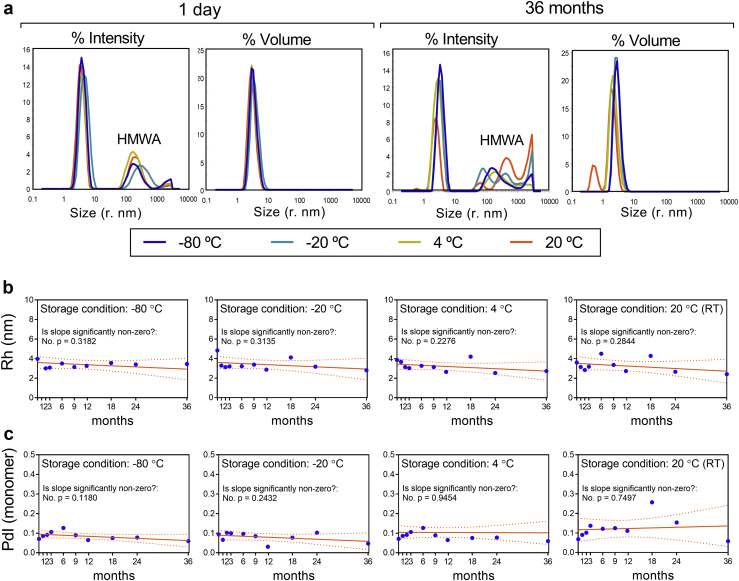
Impact of long-term storage on U-Omp19 aggregation. Samples of U-Omp19 were stored for different time periods (1 day or 1, 2, 3, 6, 9, 12, 18, 24 and 36 months) at the indicated temperatures (−80 °C, −20 °C, 4 °C, and 20 °C). The presence of aggregates and particle size were evaluated by DLS (a) Particle size distribution profiles by intensity (left), and volume (right) of samples incubated at the specified temperatures for 1 day or 36 months. Time-dependent study of the hydrodynamic radii of U-Omp19 monomer (main peak) (b) and its polydispersity index (c) for each storage temperature. Regression lines and 95% confidence intervals are shown for each condition. Fitted slopes were compared to 0 (No changes in time) using an F test and P values are depicted on each graph.

Dynamic light scattering measurements were performed to study the tendency of U-Omp19 to aggregate over time when stored at different temperatures. The intensity distribution profile for U-Omp19 showed a major peak (pk1) corresponding to the monomer protein (hydrodynamic radius: R_h_ = 4.09 ± 1.25 nm) along with a marginal presence of high molecular weight aggregates (HMWA, R_h_ > 90 nm) (Fig. 2a, left panel). However, size distribution by volume revealed the presence of a single peak (Fig. 2a, right panel), indicating that the amount of HMWA present in the sample is negligible (0% Mass). These aggregates were present at the first day (t = 1 day) and their amount did not change along the course of the study in any of the tested conditions (Supplementary Table 1). Only the sample corresponding to 36 months of storage at RT showed an increase in HMWA size, although its proportion among the entire population of particles remained insignificant. On the other side, this sample presented an additional peak (both on intensity and volume distributions) corresponding to particles of lower size, probably due to protein fragmentation (Fig. 2b). Remarkably, neither significant changes in the R_h_ nor in Polydispersity Index (PdI) of the main population over time were detected in any of the samples analyzed (Fig. 2c,d). Altogether these results indicate that aggregation is not a noteworthy degradation pathway for U-Omp19 when stored under the tested conditions for up to 3 years.

**Fig. 3:**
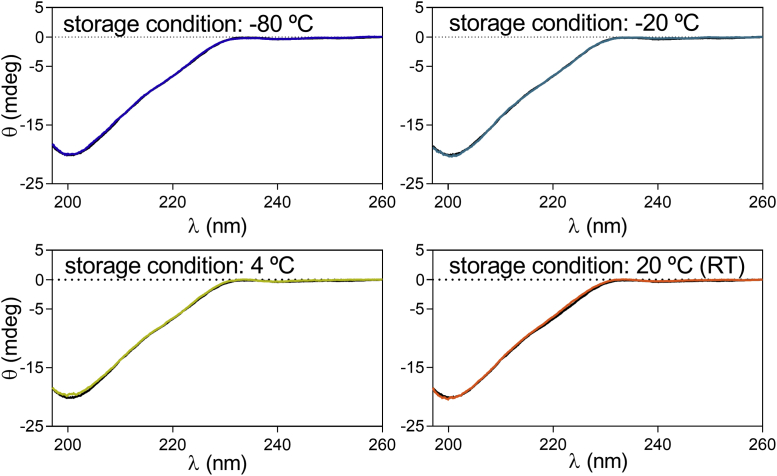
Impact of long-term storage on U-Omp19 secondary structure content. Samples of U-Omp19 were stored for up to 36 months at −80 °C, −20 °C, 4 °C, and 20 °C. The secondary structure content was evaluated by Far-UV CD. Spectra for U-Omp19 samples stored for 36 months at the indicated temperatures (color lines) compared to a fresh prepared U-Omp19 sample (black lines).

Circular Dichroism was used to monitor the effect of temperature on the overall secondary structure of U-Omp19 upon long-term storage. Interestingly, no changes in the far UV region of the CD spectra (197–260 nm) were found in the samples maintained at different temperatures during the entire study, indicating that no changes in the secondary structure of U-Omp19 had taken place (Fig. 3). Altogether, these results indicate that the secondary structure content of U-Omp19 is stable for at up to 3 years when stored in solution at −80 °C, −20 °C, 4 °C or RT.

### Proteolysis is the Major Degradation Pathway for U-Omp19 When Stored as Liquid Formulation

**Fig. 4:**
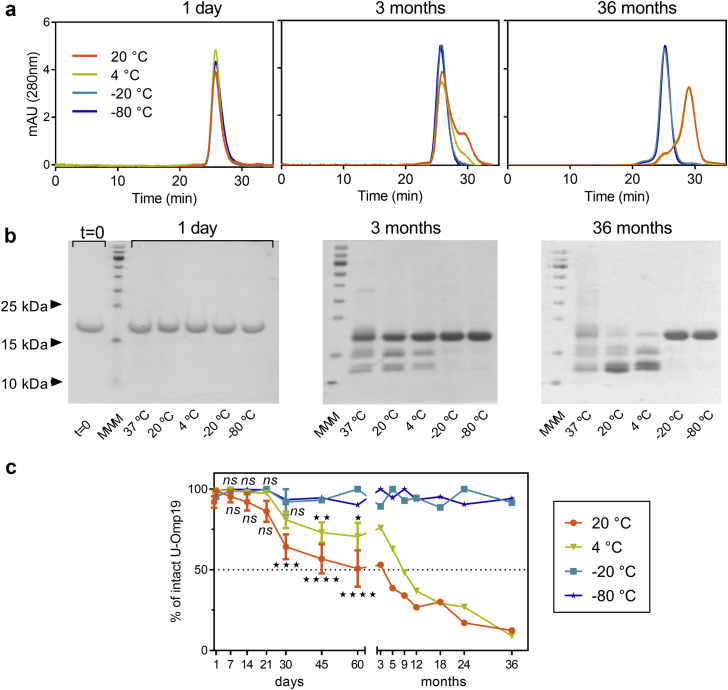
Impact of long-term storage on U-Omp19 protein integrity. Samples of U-Omp19 were stored for different time periods (1 day or 1, 2, 3, 6, 9, 12, 18, 24 and 36 months) at the indicated temperatures (−80 °C, −20 °C, 4 °C, and 20 °C). Samples were analyzed by size exclusion chromatography and SDS-PAGE. Representative size exclusion chromatography elution profiles (a) and Coomassie blue stained reduced SDS-PAGE patterns (b) of U-Omp19 stored at the specified temperatures at the indicated time points. (c) Densitometric analysis of U-Omp19 bands in SDS-PAGE using Image J. The results are shown as percentage of intact protein as a function of time for each storage condition (mean ± SEM). ★★★★: P < 0.0001, ★★★: P < 0.001, ★★: P < 0.01, ★: P < 0.05 or ns: P > 0.05 vs −80 °C condition at the same time point.

Size exclusion chromatography and SDS-PAGE analysis were performed to evaluate the integrity of U-Omp19 during storage. Elution as a unique peak in SEC and detection of a single band in SDS-PAGE revealed that samples stored at −20 °C or −80 °C retained their structural integrity and were highly stable along the 3-year study. Interestingly, upon storage at RT or 4 °C U-Omp19 underwent a marked proteolysis, which was evidenced by a second peak with higher retention time in SEC and lower molecular weight bands in SDS-PAGE (Fig. 4a,b). Statistical analysis of the kinetic of proteolysis studied by SDS-PAGE gels corroborated that the proteolytic degradation rates were significantly higher, and half-life of intact protein lower at higher storage temperature conditions (Table 1). Though, U-Omp19 samples can be stored up to 3 weeks at RT or 1 month at 4 °C without undergoing significant degradation (Fig. 4c).

**Table 1:** Kinetic Analysis of Percentage of Proteolysis.

Storage Condition	Initial Degradation Rate (% of Protein Loss/Month) (Mean ± SD)	Half-Life (Months) (Mean ± SD)	Goodness of Fit R Square
RT	−21.15 ± 3.84^a,b,c^	3.1 ± 0.4^a,b,c^	0.6413
4 °C	−7.85 ± 1.35^a,b^	8.4 ± 1.0^a,b^	0.6619
‒20 °C	−0.08 ± 0.13	783.1 ± 845.0	0.0310
‒80 °C	−0.14 ± 0.09	469.3 ± 206.2	0.0497

Data plotted in Fig. 4c (% of undigested protein versus time) were fitted to one phase decay equation model to compare the behavior of samples. Best fit values were used to estimate the initial rates of degradation and the half-life for each condition. The goodness of fit is depicted as R square. For frozen storage conditions, the proteolysis was not significant and thus the goodness of fit was low. An extra sum-of-squares F test was applied to compare if fitted parameters are statistically different for each condition: a: P < 0.001 vs. −80 °C condition; b: P < 0.001 vs. −20 °C condition; c: P < 0.001 vs. 4 °C condition.

In order to investigate whether U-Omp19 fragmentation could be due to trace protease contamination, additional work was conducted to screen excipients or sample treatments that may improve the stability of U-Omp19 in the proposed buffer formulation (50 mM phosphate, 300 mM sodium chloride at pH 7.4) (Table 2). Neither the addition of a second purification step (SEC), nor sample treatment with the serine protease inhibitor PMSF, the metal chelating agent EDTA or heat were able to reduce the observed proteolysis, suggesting that it may be caused by a non-enzymatic mechanism (Supplementary Fig. 2a-c). In addition, since it is known that buffer composition and pH can affect non-enzymatic proteolysis,^14^^,^^21^^,^^22^ U-Omp19′s samples were exchanged into buffers detailed in Table 2 and stored for 1 month at −80 °C or RT. Extent of cleavage of U-Omp19 samples was followed by SDS-PAGE. The results showed that storage in buffers with lower pH increased the rate of proteolysis and no significant reduction in proteolysis was observed when stored at neutral or basic pH conditions (HEPES, Saline or Tris buffer) (Supplementary Fig. 2d).

**Table 2:** Samples of U-Omp19 in the Indicated Buffers with the Addition of the Indicated Additives or Subjected to the Indicated Treatments were Incubated at 20 °C or 37 °C (Accelerated Forced Degradation Condition).

Incubation Buffer:	50 mM Sodium Phosphate, 300 mM Sodium Chloride, pH 7.4	
	Additives	None
		1 mM PMSF
		5 mM EDTA
	Treatment	None
		Heating (20 min, 80 °C) Second purification step (SEC)
Incubation buffer:	50 mM sodium phosphate, 300 mM sodium chloride, pH 4.0	
	20 mM sodium citrate, 100 mM sodium chloride pH 5.5	
	20 mM Hepes buffer, 100 mM sodium chloride pH 7.4	
	20 mM Tris Buffer, 100 mM sodium chloride pH 7.4	
	150 mM sodium chloride, pH 7.0 (saline)	

Altogether these long-term storage studies indicate that U-Omp19 is stable when stored up to 36 months at −20 °C or −80 °C, 1 month at 4 °C or 3 weeks at RT. Under these specific conditions, the protein adjuvant retains its ability to inhibit proteases without undergoing substantial proteolysis, aggregation or changes in its secondary structure.

### U-Omp19 is Stable Upon Repeated Freeze-Thaw Cycles

**Fig. 5:**
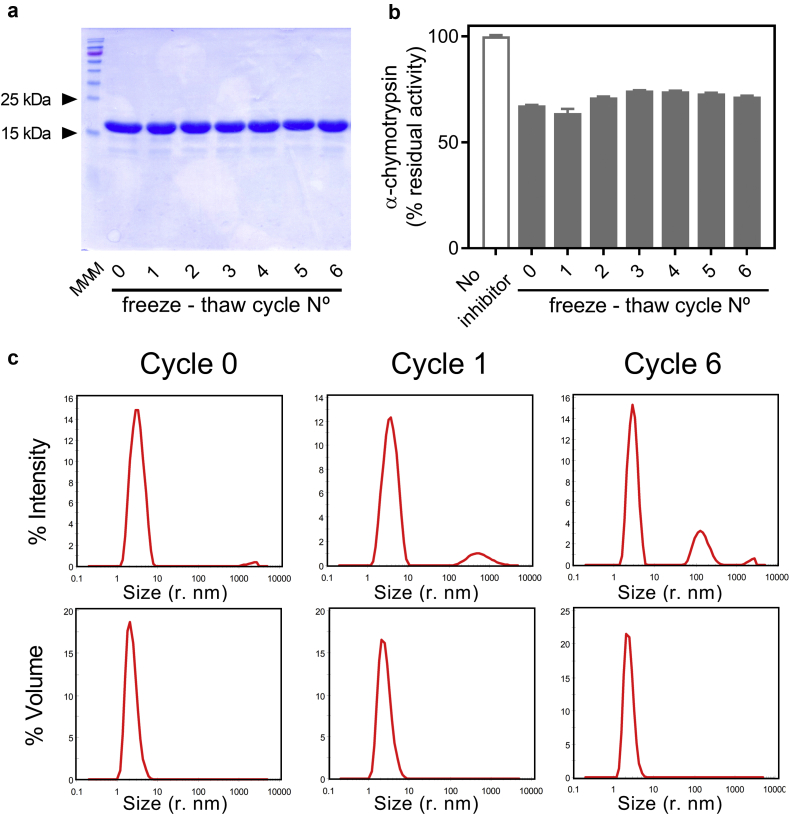
Impact of multiple freeze-thaw cycles on U-Omp19 stability. Samples of U-Omp19 were subjected to multiple freeze and thaw cycles. (a) SDS–PAGE Coomassie stained gels of non-reduced U-Omp19 samples after 0 (lane 2) to 6 (lane 8) freeze-thaw cycles. Lane 1: Molecular Weight Markers (MWM). (b) Percentage of α-chymotrypsin proteolytic activity remaining upon incubation with buffer (No inhibitor) or 50 μM of each U-Omp19 sample after 0 to 6 freeze-thaw cycles. (c) Dynamic light scattering measurements: Representative particle size distribution profiles by intensity and volume for U-Omp19 samples without freezing (Cycle 0) or after 1 or 6 freeze-thaw cycles.

Proteins are purposely frozen for storage. Freezing generally slows protein degradation; however, it is not devoid of risks since the physical environment of the protein changes dramatically during the process, leading to the development of stresses that impact protein stability. We aimed to examine the U-Omp19`s stability following numerous freeze-thaw cycles. U-Omp19 samples neither formed precipitates, nor showed changes in protein concentration after repeated freeze and thaw cycles (data not shown). Remarkably, protein integrity was conserved (Fig. 5a) and U-Omp19 retained its full inhibitory activity after being frozen and thawed up to 6 times (Fig. 5b). Moreover, freeze-thaw cycles didn't induce significant protein aggregation (Fig. 5c). Together these results indicate that U-Omp19 can afford multiple cycles of freeze and thaw without undergoing significant degradation, maintaining its physicochemical and biological properties unchanged.

### Lyophilization (Freeze Drying) of U-Omp19 Overcomes Fragmentation and Allows Room Temperature Storage for at Least 18 Months But Induces Protein Aggregation

**Fig. 6:**
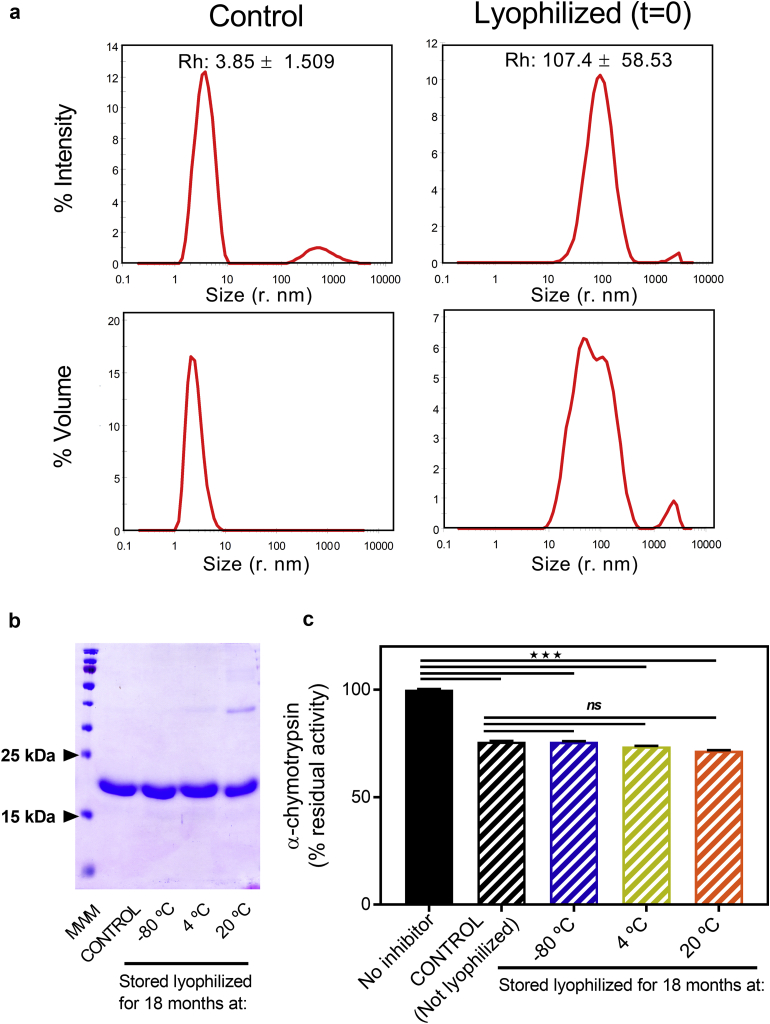
Impact of lyophilization and long-term storage in solid state on protease inhibitor activity, protein integrity, and aggregation of U-Omp19. Samples of U-Omp19 were freeze dryed and placed for long term stability studies for up to 18 months at −80 °C, 4 °C, and 20 °C (RT). (a) Dynamic light scattering measurements: Comparison of particle size distribution profiles by intensity and volume for U-Omp19 samples without freeze drying (Control) or after lyophilization and instant reconstitution (t = 0). (b) SDS–PAGE Coomassie stained gel of reduced U-Omp19 samples. Lane 1: Molecular Weight Markers (MWM), Lane 2: U-Omp19 in aqueous solution (not lyophilized). Lane 3 to 5: lyophilized U-Omp19 after reconstitution stored in solid state for 18 months at the indicated temperatures. (c) Recovered inhibitor activity expressed as percentage of α-chymotrypsin proteolytic activity (mean ± SEM) remaining upon incubation with buffer (No inhibitor), 50 μM of U-Omp19 control sample (not lyophilized) or lyophilized and rehydrated U-Omp19 samples after storage in solid state for 18 months at indicated temperatures. ★★★: P < 0.001 vs No inhibitor condition and ns: P > 0.05 vs non-lyophilized condition.

Since protein fragmentation is the main degradation pathway that U-Omp19 undergoes when stored at room temperature, lyophilization may be a useful process to stabilize it and potentially extend its shelf life alone or as part of vaccine formulations as well.^23^ Thus, we subsequently studied the effect of lyophilization and storage on U-Omp19`s activity, integrity and aggregation tendency. Samples of U-Omp19 were lyophilized and stored conveniently at −80 °C, 4 °C or RT for up to 18 months. Lyophilized U-Omp19's samples were easily, completely and quickly reconstituted when mixed with high purity deionized water. Neither protein loss nor visible particulates were observed after reconstitution (data not shown). However, DLS analysis of reconstituted samples revealed the presence of significant HMWA. Distribution by intensity and volume showed that mostly all the protein was forming HMWA (Fig. 6a). Nevertheless, non-reducing SDS-PAGE showed that the nature of these aggregates was predominantly non-covalent, and that protein integrity was conserved in lyophilized samples stored at RT or in the fridge for up to 18 months (Fig. 6b). Furthermore, U-Omp19 protease inhibitor activity upon lyophilization, storage and reconstitution remained unaltered compared to the activity of non-lyophilized samples (control) (Fig. 6c). These results together indicate that although aggregation appears to be the major degradation pathway in U-Omp19's lyophilized samples, lyophilization abrogates the proteolytic degradation of U-Omp19 and allows protein stabilization even at RT for at least 18 months without undergoing protease inhibitor activity loss. In any case, ongoing formulation studies will significantly contribute to the development of buffer conditions that mitigate the impact of lyophilization on U-Omp19 aggregation.

## Discussion

A prerequisite for the production of safe protein-based subunit vaccines and adjuvants is to avoid changes in its chemical, physical, microbiological and biological properties throughout its shelf-life. That may reduce potency, limit shelf life, and could increase the potential for undesired side effects.^17^^,^^24^ The identification of intrinsic and extrinsic factors that contribute to the stabilization of proteins has provided valuable information for stabilizing protein pharmaceuticals and for designing more stable mutant proteins. Yet the structural differences among different proteins are so significant that generalization of universal stabilization strategies has not been successful. Very often, proteins have to be evaluated individually and stabilized on a trial-and-error basis.^14^ Determining instabilities of proteins remains to be one of the major road barriers, hindering rapid commercialization of potential protein drug candidates, including protein based vaccine adjuvants.^25^ A comprehension of protein stability is essential for optimizing the expression, purification, formulation, and storage of protein products. The development and eventual successful commercial manufacturing of a well-defined, stable, scalable production process of a clinically efficacious protein vaccine adjuvant (U-Omp19) will depend in part on a detailed mechanistic understanding of the protein's physicochemical properties, integrity and stability as well as the interrelationships between these structural properties and functional attributes. In this work, the degradation behaviors of U-Omp19 were investigated through a comprehensive assessment including the analysis of its protease inhibitor activity, conformational stability, aggregation tendency and protein integrity.

The stability of protease inhibitor activity of U-Omp19 is a critical factor to be determined, since this activity has been suggested to be linked to the increase of antigen amount that reaches the inductive sites of the mucosa in the gut and the increase of antigen cross presentation by dendritic cells.^9^^,^^10^^,^^13^ Protease inhibitor activity of U-Omp19 has been shown to be both thermal and pH stable.^10^ Here we have demonstrated that U-Omp19 retains its full protease inhibitor activity when stored in solution at different temperatures up to 36 months (Fig. 1). This long-term stability of U-Omp19 is similar to other known protease inhibitors, for example Aprotinin, a bovine protein that is stable at least for one month at 4 °C and below −20 °C for years,^26^ or the trypsin inhibitor from Glycine max (Soybean) Type I–S A that can be stored for 3.5 years at 2–8 °C without loss of inhibition activity.^27^

Protein aggregation is arguably the most common and troubling manifestation of protein instability, encountered in almost all stages of protein drug development.^25^^,^^28^ Protein aggregation is known to reduce yields and affect the potency of a number of vaccine antigens.^29^, ^30^, ^31^ For example, the mucosal adjuvant dmLT is highly prone to aggregation during agitation stress with different-sized aggregates formed ranging from 1 nm to >100 μm and a concomitant loss of protein content.^32^ Therefore, the characterization of protein aggregates and controlling their formation is a critical step during vaccine development. Interestingly, U-Omp19 didn't form aggregates when stored in solution in laboratory freezers (−20 °C or −80 °C), fridge (4 °C) or at RT up to 36 months (Fig. 2). This attribute may be an advantage for U-Omp19's formulation development, since several antigens and adjuvants have been shown to aggregate upon freezing or during storage at 2–8 °C or at RT, i.e. seasonal influenza vaccines.^31^ Although the presence of HMWA was detected using DLS (Fig. 2), the amount of these aggregates was insignificant and did not change over time in the storage conditions tested. Only a smaller size peak was detected after 36 months of storage at RT that may be related to the proteolytic events that occurs during storage. Unfolding of secondary and tertiary structure is the first step of denaturation processes that exposes hydrophobic residues and leads to aggregation.^33^ In agreement with DLS results, Far UV CD studies (Fig. 3) showed no changes in overall secondary structure content. Additionally, SEC (Fig. 4a) and SDS-PAGE (Fig. 4b) results ruled out the presence of aggregates, since neither species with lower retention time in SEC profile nor higher molecular weight bands in SDS-PAGE were evidenced along this study. Nevertheless, since samples were centrifuged and no other assessments of protein precipitation or particle formation than visual inspection were performed prior to centrifugation of samples, small quantities of particle formation over time could be underestimated if they remained subvisible. In general, low levels of soluble aggregates in pharmaceutical products can be tolerated as long as the product remains stable, and soluble aggregates do not progress to insoluble forms.^25^

Despite the importance of physical stability in determining the long-term stability of protein pharmaceuticals, protein integrity has to be insured for a proper protein drug formulation as well. Our results indicate that U-Omp19 is stable up to 36 months when stored frozen, or as liquid solution for 1 month at 4 °C or three weeks at RT. However, it undergoes significant fragmentation after 30 days of storage at RT or 45 days at 4 °C. Fragmentation refers to disruption of a covalent bond in a protein as a result of either spontaneous or enzymatic reaction. The protein backbone is extremely stable under physiological conditions, but certain sites may become prone to fragmentation as a function of amino acid sequence (presence of specific side-chains that may facilitate cleavage), flexibility of the local structure, solvent conditions (pH, temperature) and the presence of metals or radicals.^14^^,^^28^ U-Omp19's fragmentation appears to be a kinetic process that is not caused by low levels of proteases or catalyzed by transition metals since addition of EDTA, protease inhibitors or heating have no effect on U-Omp19 hydrolysis rates (Supplementary Fig. 2a-c). This result is similar to hinge region hydrolysis described for different human IgG1s, that typically occurs in the flexible regions and the hydrolysis reaction is not specific to a particular peptide bond but occurs within a narrow range of residues.^21^^,^^22^ Another critical formulation condition is pH, that rules protein stability by controlling the protein's surface net charge, density and distribution, and may influence both the conformational and structural stability.^34^ U-Omp19 retains its secondary structure and its full protease inhibitor activity when exposed to a wide range of pH and temperature conditions, indicating that is pH- and thermo-stable.^10^ However, likewise to non-enzymatic hinge region hydrolysis of human IgGs,^35^^,^^36^ proteolysis of U-Omp19 during long term storage in solution is accelerated when stored at low pH buffers (Supplementary Fig. 2d). Notwithstanding U-Omp19′s proteolysis, Far-UV CD spectra and protease inhibitor activity remained unaltered, indicating that the fragments present together contribute additively to the secondary structure content and to protease inhibitor activity of this protein. Together these results will shed light into ongoing studies of our laboratory on structure-activity relationship of U-Omp19. A better understanding of the structure-activity relationship of U-Omp19's adjuvanticity, may allow mutations and modifications that could be introduced (e.g., site-directed mutagenesis) to improve its inherent stability.

To achieve an overall stable formulation is the main target in any protein/drug formulation process. Protein pharmaceuticals usually must be stored under cold conditions or even freeze-dried to a solid form to achieve an acceptable shelf life. Physical instability due to heat and/or freeze stress of antigens or adjuvants can be a major cause of potency loss in various vaccines during manufacturing, storage and administration even within the vaccine cold chain. Frozen storage is an important preservation method for proteins.^37^^,^^38^ However, the freezing process and frozen storage can affect the quality of the product.^39^ Vaccines containing aluminum salt adjuvants are prone to inactivation following exposure to freeze-thaw stress.^40^^,^^41^ Some proteins may undergo unfolding, aggregation or changes in concentration over time in the frozen state.^34^^,^^38^^,^^39^^,^^42^ In this work, U-Omp19, was shown to retain its full inhibitory activity, folding, integrity, while no significant aggregation was induced when subjected to freezing (Fig. 5).

Freeze-thaw is often explored as a forced degradation condition to determine the susceptibility of a protein to temperature cycling, since low temperature, freeze-concentration, and ice formation damages may occur during the process. Freeze–thawing is a common stress to which a protein drug can be exposed to deliberately or accidently, once or multiple times during manufacturing, shipping, and storage and may lead to protein denaturation, precipitation, aggregation, and loss of function.^38^^,^^43^, ^44^, ^45^, ^46^, ^47^ Proteolysis is in general not the major degradation pathway for freeze-thaw stress because of the short time exposure of protein to RT.^48^ Accordingly, U-Omp19 can be subjected up to 6 cycles of freeze and thaw without undergoing proteolysis (Fig. 5). During freeze and thaw cycles the protein is subjected to changes in temperature, concentration, pH and ionic strength.^42^ The pH of phosphate-buffered solutions is temperature-dependent which can lead to protein instability during freeze-thaw cycles. Despite this fact, no instability of U-Omp19 was observed during temperature cycling, which indicates that U-Omp19 remain physically stable under these pH conditions in agreement with previous work showing that this protein is stable in a wide range of pH.^10^ A second factor is that sodium chloride concentrates during freezing, which can increase the ionic strength of solutions.^42^ But again, as we didn't see physical changes in these studies, we can infer U-Omp19 remains physically stable under these ionic strength fluctuations. Proteins subjected to repeated freeze/thaw cycles may lose activity, however here we have demonstrated that the inhibitor activity of U-Omp19 can resist repetitive freeze and thaw cycles (Fig. 5b). The major degradation pathway during freeze-thaw is frequently the formation of various aggregates, including precipitates and particles.^48^, ^49^, ^50^ Despite high molecular weight aggregates were detected after 1 or 6 cycles of freeze and thaw, the amount of these aggregates was insignificant. Similar to dmLT in a recent newly developed formulation,^50^ U-Omp19 is freeze-thaw stable for up to six freeze/thaw cycles with no significant aggregation nor loss of protein observed (Fig. 5). Nevertheless, the addition of a cryoprotectants and formulation optimization like was applied by Toprani et al. for dmLT^50^ would be useful to avoid marginal protein aggregation during freeze and thaw of U-Omp19.^50^ Our results highlight that U-Omp19 is very stable to freeze and thaw cycles, this attribute would be of great interest since vaccine manufacturers have long been interested in formulations that can withstand freezing.^40^^,^^50^^,^^51^

Most protein drugs and also some vaccine components are freeze-dried to improve their storage stability, i.e. the mucosal adjuvant dmLT.^50^ Freeze drying is often used to increase the stability and shelf life of protein drugs and is frequently used to prevent chemical and physical degradation.^24^^,^^42^ Moreover, storage and distribution are cost-reduced and simplified, mitigating cold-chain requirements. However, a variety of stresses are encountered during the freeze-drying steps (low temperature, phase separations, pH and ionic strength changes, and ice crystal formation among others) which may lead to protein unfolding or degradation.^34^^,^^42^ Frequently after lyophilization loss of protein or inability to redissolve occurs, indicating denaturation during the process. Interestingly, reconstitution with high purity deionized water of lyophilized U-Omp19's samples was quick, and no visible particles nor protein loss were detected afterwards. The major change for U-Omp19 after lyophilization is aggregation, since almost all protein present in the samples formed non-covalent HMWA after reconstitution (Fig. 6). Remarkably, formation of these aggregates does not alter the inhibitor activity of U-Omp19. However, as aggregate formation has been linked to vaccine potency loss as well as to safety concerns,^29^^,^^30^ future work improving the lyophilization steps and/or excipients composition is needed to minimize the aggregation of U-Omp19 upon lyophilization as part of formulation development.^34^^,^^38^ Furthermore, our results indicate that lyophilization allows U-Omp19 storage at RT up to 18 months without undergoing fragmentation nor loosing protease inhibitor activity.

In this work we performed a preformulation characterization of U-Omp19, determining its stability along different storage conditions. At the same time, we have developed biophysical techniques and activity-based assays to study U-Omp19 stability. An understanding of the physicochemical properties of U-Omp19 using the stability-indicating analytical methods resulted from this work will contribute to the development of a more rational formulation and an optimized process design that will improve storage stability, both as a frozen bulk substance and eventually as a final dosage form of a vaccine containing this novel adjuvant.

## Author Contributions Statement

MLD: Experimental design, Methodology, Data interpretation and Writing. MLC: Experimental design, Methodology, Supervision, Data interpretation and Reviewing. LB: Methodology. JC: Conceptualization, Funding, Experimental design, Supervision and Reviewing. KAP: Conceptualization, Funding, Experimental design, Supervision, Data analysis, Original draft preparation and Writing.

## Conflicts of Interest

All authors: No reported conflicts of interest.
