# Supplementary material for: Stability Studies of the Vaccine Adjuvant U-Omp19
Source: J Pharm Sci. 2021 Feb;110(2):707–18. doi: 10.1016/j.xphs.2020.10.011 (PMC7815325; doi:10.1016/j.xphs.2020.10.011)
Supplement: Multimedia component 1 [file mmc1.docx]

**Supplementary Material.**

**Supplementary Figure 1.**


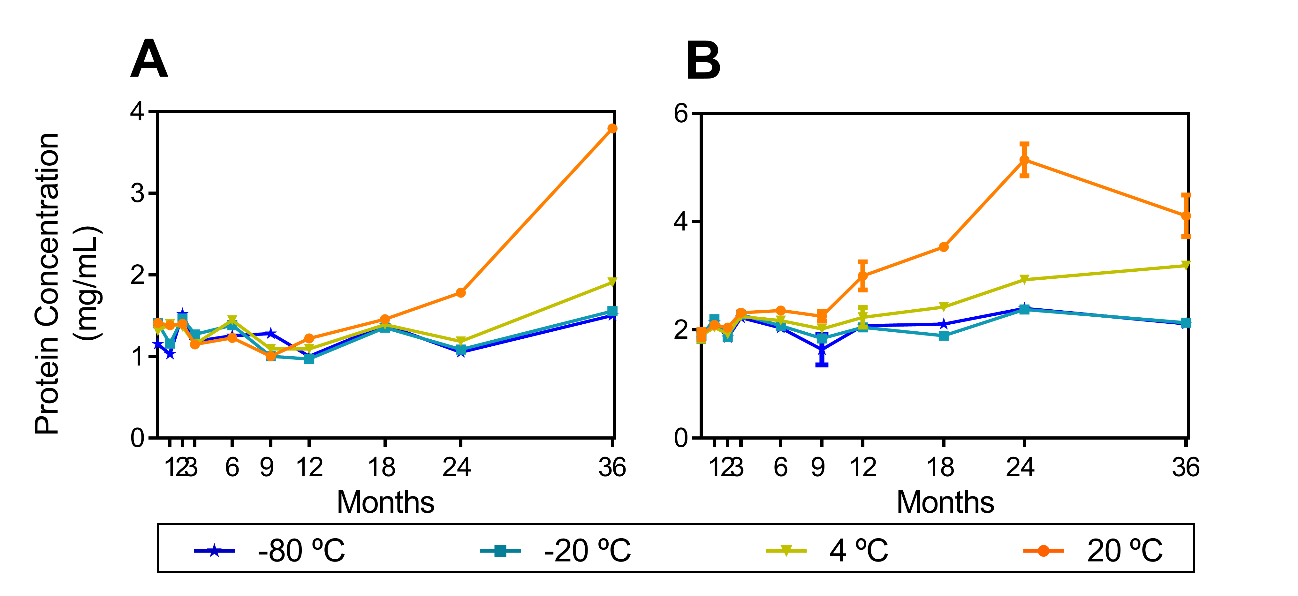


**Supplementary Figure 1. Impact of long-term storage on U-Omp19 concentration**. Samples of U-Omp19 were stored for different time periods (1 day or 1, 2, 3, 6, 9, 12, 18, 24 and 36 months) at the indicated temperatures (‒80 °C, ‒20 °C, 4 °C, and 20 °C). Protein sample concentrations were quantified spectrophotometrically (**A**) and by BCA (**B**). Results are shown as protein concentration as a function of time for each storage condition. Reported values were the average of 3 different measurements for panel A, and mean ±SD of duplicate measurements for panel B.

**Supplementary Table 1. Analysis of the intensity and volume distribution of the major size population present in U-Omp19 samples.**

|  |  | Pk 1 | | | |
| --- | --- | --- | --- | --- | --- |
| t | Storage Temperature | Rh (nm) | PdI | % Intensity | % Mass |
| 1 day | RT | 3.605 ± 0.9402 | 0.0681 | 69.5 | 100.0 |
|  | 4 °C | 3.875 ± 1.0150 | 0.0686 | 62.1 | 100.0 |
|  | -20 °C | 4.840 ± 1.4830 | 0.0936 | 74.4 | 99.9 |
|  | -80 °C | 3.987 ± 1.0590 | 0.0708 | 72.8 | 100.0 |
| 1 month | RT | 3.129 ± 0.9374 | 0.0900 | 73.7 | 100.0 |
|  | 4 °C | 3.701 ± 1.0950 | 0.0876 | 69.2 | 100.0 |
|  | -20 °C | 3.281 ± 0.8432 | 0.0660 | 65.4 | 100.0 |
|  | -80 °C | 4.159 ± 1.2250 | 0.0864 | 74.8 | 100.0 |
| 2 months | RT | 2.844 ± 0.9036 | 0.1011 | 84.2 | 100.0 |
|  | 4 °C | 3.139 ± 1.1370 | 0.1310 | 86.8 | 100.0 |
|  | -20 °C | 3.125 ± 1.0020 | 0.1024 | 83.3 | 99.9 |
|  | -80 °C | 3.009 ± 0.9081 | 0.0912 | 71.9 | 100.0 |
| 3 months | RT | 3.180 ± 1.1770 | 0.1369 | 96.9 | 100.0 |
|  | 4 °C | 3.032 ± 1.0070 | 0.1102 | 83.1 | 100.0 |
|  | -20 °C | 3.194 ± 1.0110 | 0.0999 | 79.3 | 100.0 |
|  | -80 °C | 3.086 ± 1.0070 | 0.1063 | 81.0 | 100.0 |
| 6 months | RT | 4.523 ± 1.5770 | 0.1218 | 90.0 | 100.0 |
|  | 4 °C | 3.256 ± 1.1350 | 0.1218 | 93.6 | 100.0 |
|  | -20 °C | 3.215 ± 1.0010 | 0.0967 | 89.7 | 100.0 |
|  | -80 °C | 3.499 ± 1.2450 | 0.1267 | 81.1 | 100.0 |
| 9 months | RT | 3.356 ± 1.1840 | 0.1246 | 83.0 | 100.0 |
|  | 4 °C | 3.126 ± 1.0210 | 0.1069 | 80.3 | 100.0 |
|  | -20 °C | 3.369 ± 0.9810 | 0.0847 | 76.4 | 100.0 |
|  | -80 °C | 3.144 ± 0.9407 | 0.0894 | 77.0 | 100.0 |
| 12 months | RT | 2.739 ± 0.9135 | 0.1109 | 88.6 | 100.0 |
|  | 4 °C | 2.641 ± 0.6724 | 0.0650 | 90.0 | 100.0 |
|  | -20 °C | 2.869 ± 0.5062 | 0.0310 | 50.0 | 100.0 |
|  | -80 °C | 3.256 ± 0.8302 | 0.0650 | 66.7 | 100.0 |
| 18 months | RT | 4.303 ± 2.1810 | 0.2570 | 85.4 | 100.0 |
|  | 4 °C | 4.277 ± 1.7920 | 0.1681 | 77.0 | 100.0 |
|  | -20 °C | 4.106 ± 1.1420 | 0.0773 | 71.3 | 100.0 |
|  | -80 °C | 3.544 ± 0.9742 | 0.0756 | 61.1 | 100.0 |
| 24 months | RT | 2.649 ± 1.039 | 0.1537 | 84.7 | 100.0 |
|  | 4 °C | 2.540 ± 0.7760 | 0.0936 | 80.2 | 100.0 |
|  | -20 °C | 3.175 ± 1.0150 | 0.1024 | 75.1 | 100.0 |
|  | -80 °C | 3.416 ± 0.9533 | 0.0778 | 72.0 | 100.0 |
| 36 months | RT | 2.412 ± 0.5812 | 0.0581 | 37.7 | 81.9 |
|  | 4 °C | 2.852 ± 0.8254 | 0.0835 | 69.0 | 100.0 |
|  | -20 °C | 3.190 ± 0.6930 | 0.0471 | 51.6 | 99.9 |
|  | -80 °C | 3.459 ± 0.8434 | 0.0595 | 65.1 | 100.0 |
| Pk1: Major peak in the intensity and volume distribution. Rh: hydrodynamic radius. PdI: polydispersity index. % Intensity: Percentage area in the intensity distribution. % Mass: Percentage area in the volume distribution. Rh values are expressed as Mean ± SD. | | | | | |

**Supplementary Figure 2.**


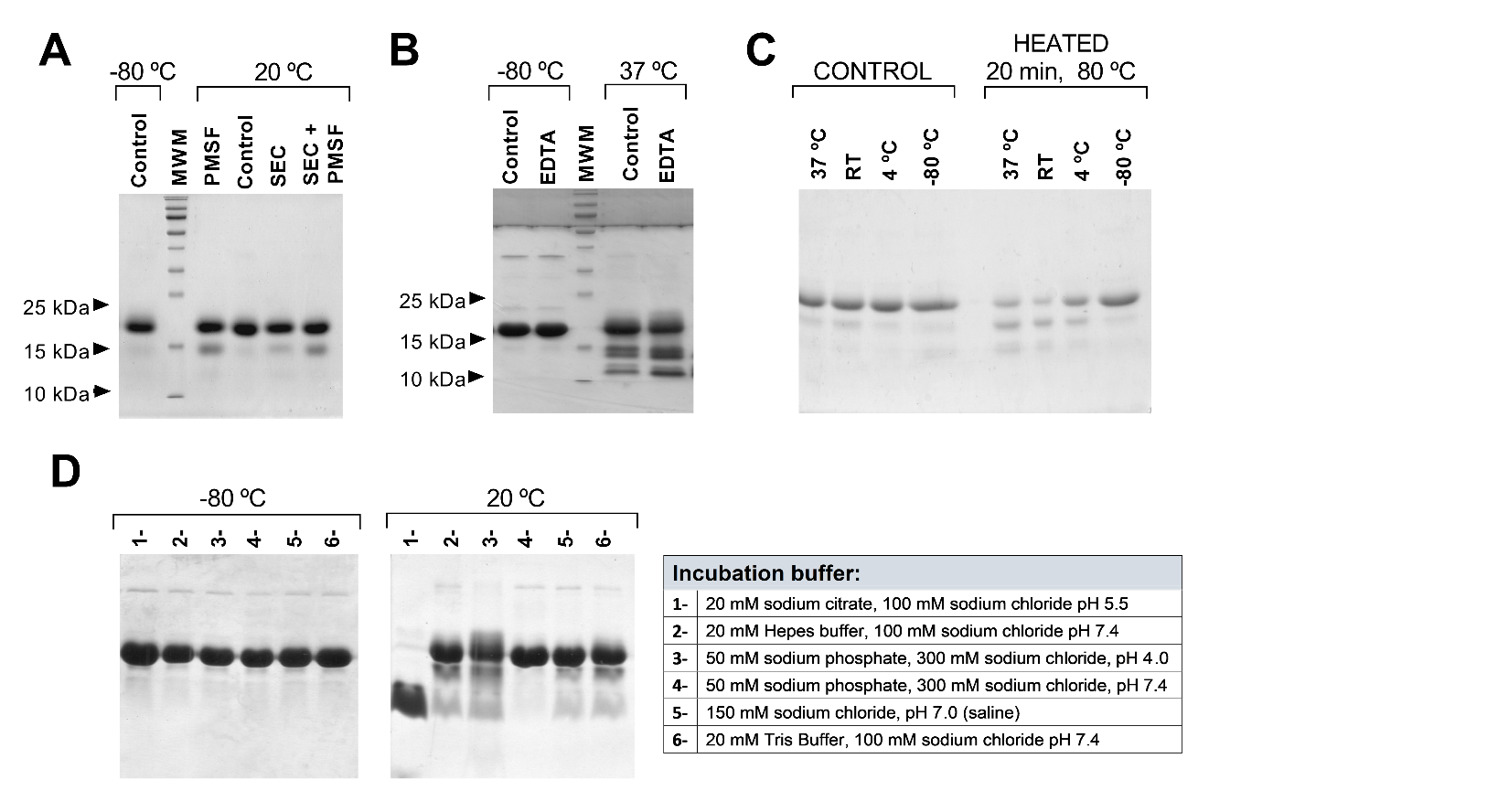


**Supplementary Figure 2. Analysis of buffer composition, treatments and additives in U-Omp19´s fragmentation during storage as liquid formulation.** Integrity of non reduced U-Omp19 was monitored by SDS-PAGE in samples stored for 1 month either at ‒80 °C, 20 °C, or 37 °C (accelerated forced degradation condition) with the addition of the indicated additives, subjected to the specified treatments, or exchanged into the detailed buffers. Impact of: (A) the addition of a serine-protease inhibitor (PMSF 1 mM), and/or with a second purification step (SEC), (B) the addition of a metalloproteinase inhibitor and chelator (EDTA 5 mM), (C) heating for 20 min at 80 ºC, or (D) buffer composition was analyzed on the cleavage rates of U-Omp19. Gels were compared visually to determine if they are comparable to the reference standard. At least 3 different lots of U-Omp19 were studied for each condition.
